# Anthraquinone-Quinizarin Copolymer as a Promising Electrode Material for High-Performance Lithium and Potassium Batteries

**DOI:** 10.3390/molecules28145351

**Published:** 2023-07-12

**Authors:** Elena V. Shchurik, Olga A. Kraevaya, Sergey G. Vasil’ev, Ivan S. Zhidkov, Ernst Z. Kurmaev, Alexander F. Shestakov, Pavel A. Troshin

**Affiliations:** 1 Federal Research Center for Problems of Chemical Physics and Medicinal Chemistry RAS, 1 Prospekt Akademika Semenova, 142432 Chernogolovka, Russiaokraevaya@inbox.ru (O.A.K.);; 2 Higher Chemical College of RAS, D.I. Mendeleev University of Chemical Technology of Russia, 9 Miusskaya square, 125047 Moscow, Russia; 3 Institute of Physics and Technology, Ural Federal University, Mira 19 Str., 620002 Yekaterinburg, Russia; i.s.zhidkov@urfu.ru (I.S.Z.); ernst.kurmaev@gmail.com (E.Z.K.); 4 M.N. Mikheev Institute of Metal Physics of Ural Branch of Russian Academy of Sciences, S. Kovalevskoi 18 Str., 620108 Yekaterinburg, Russia; 5 Faculty of Fundamental Physics & Chemical Engineering, Lomonosov Moscow State University, GSP 1, 1-51 Leninskie Gory, 119991 Moscow, Russia; 6 Zhengzhou Research Institute, Harbin Institute of Technology, Longyuan East 7th 26, Jinshui District, Zhengzhou 450003, China; 7 Harbin Institute of Technology, No.92 West Dazhi Street, Nan Gang District, Harbin 150001, China

**Keywords:** organic cathode, lithium ion battery, potassium ion battery

## Abstract

The growing demand for cheap, safe, recyclable, and environmentally friendly batteries highlights the importance of the development of organic electrode materials. Here, we present a novel redox-active polymer comprising a polyaniline-type conjugated backbone and quinizarin and anthraquinone units. The synthesized polymer was explored as a cathode material for batteries, and it delivered promising performance characteristics in both lithium and potassium cells. Excellent lithiation efficiency enabled high discharge capacity values of >400 mA g^−1^ in combination with good stability upon charge–discharge cycling. Similarly, the potassium cells with the polymer-based cathodes demonstrated a high discharge capacity of >200 mAh g^−1^ at 50 mA g^−1^ and impressive stability: no capacity deterioration was observed for over 3000 cycles at 11 A g^−1^, which was among the best results reported for K ion battery cathodes to date. The synthetic availability and low projected cost of the designed material paves a way to its practical implementation in scalable and inexpensive organic batteries, which are emerging as a sustainable energy storage technology.

## 1. Introduction

Metal ion batteries are currently a dominant electrochemical energy storage technology, especially for portable electronics [1]. A metal ion battery is a complex electrochemical system which usually incorporates both organic and inorganic materials. The cathode is one of the most expensive and actively investigated components of the metal ion battery [2]. Conventional inorganic cathode materials based on salts and oxides of transition metals not only require an energy-intense production process, but also may lead to environmental pollution if not properly recycled. Additionally, the vast majority of the reported inorganic cathode materials do not meet the requirements for modern batteries, such as a fast charge/discharge capability in combination with high energy density [3].

Organic cathode materials have obvious advantages compared to their inorganic counterparts: they are based on light, abundant elements (e.g., C, H, O, N, S) and can be recycled as common household waste [4]. By means of modern synthetic chemistry, organic materials can be easily designed to reach high discharge capacities and required discharge potentials. Additionally, organic materials have a soft, non-crystalline structure and simple charge–discharge mechanisms which make them perfectly compatible with abundant metals beyond lithium, including sodium, potassium, zinc, and aluminum, and they enable efficient operation under high current densities, which is generally impossible for inorganic crystalline electrode materials [2]. 

**Figure 1:**
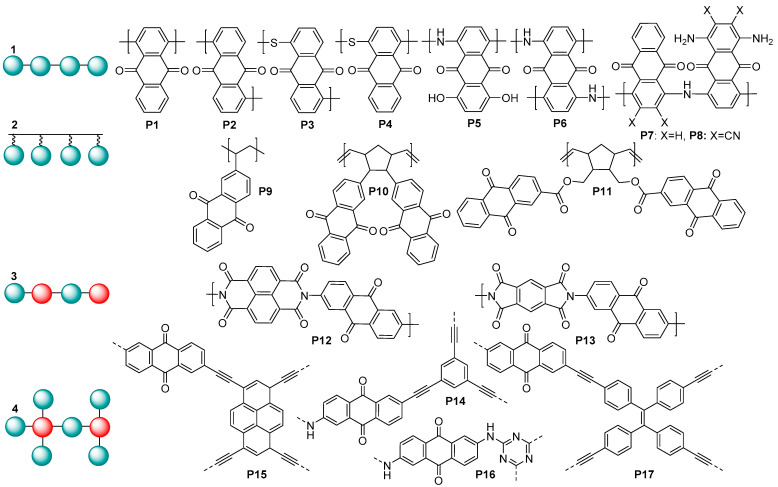
Structures of the previously studied anthraquinone-based redox-active polymers.

A wide range of organic small molecules and polymers have been reported as cathode materials for lithium and potassium ion batteries (LIBs and PIBs), including arylamines, carboxylic acids, amides, imines, and carbonyl and nitroxyl radical-derived compounds. The first attempts to utilize a carbonyl compound, namely dichloroisocyanuric acid, as an organic electrode material can be traced back to as early as 1969 [5]. Due to their intrinsically fast kinetics and high capacity, carbonyl compounds still represent one of the most actively investigated families of organic redox-active materials [6], mostly in polymeric form to prevent solubilization of the active material in the electrolytes. Among a wide range of polymeric carbonyl compounds, anthraquinone (AQ)-based polymers were reported as being highly promising electrode materials for metal ion batteries (Figure 1).

Generally, AQ-derived polymers can be divided into several groups (Figure 1) based on their chemical structures: (1) linear anthraquinones with a conjugated core; (2) polymers obtained via polymerization of the substituent fragment (e.g., alkene); (3) linear copolymers of anthraquinones with other redox-active fragments; and (4) covalent organic frameworks. When performing molecular design of the electrochemically active polymers for metal ion batteries, a series of criteria have to be taken into account: high theoretical capacity, suitable charge–discharge potentials, high conductivity, low solubility, high stability under cycling conditions, and general simplicity of synthesis. Considering these guidelines, type (1) linear anthraquinones seem to represent one of the most promising groups of AQ-based polymers due to the lower content of redox-inactive ballast weight and the presence of a conjugated core, which can potentially improve conductivity. 

Anthraquinone-based polymers have been actively utilized as electrode materials for lithium, sodium, and potassium ion batteries; for the fabrication of symmetric all-organic aqueous batteries [7]; for all-solid-state lithium ion batteries [8]; and for several other types of batteries. In lithium batteries, the linear polymers **P1** and **P2** at a 0.2C current rate showed the specific capacities of 263 and 240 mAh g^−1^, respectively, which are close to the theoretical values; in addition, a very small voltage gap between the charge and discharge curves and a stable cycling performance were obtained along with a fast discharge/charge capability [9]. When utilized as a cathode in LIBs, **P5** demonstrated a capacity of 143 mAh g^−1^ and showed an improved cycle performance compared to its monomer [10]. **P6–P8** have been investigated as cathode materials in half cells with a potassium anode. They delivered high K storage capacities (160–185 mAh g^−1^) and good cycling stabilities (up to 200 cycles) [11]. **P9** was used as an electrochemically active material in high-density rechargeable polymer/air batteries [12]. In polymer–air secondary batteries, redox-active polynorbornene **P10** showed a good cycle performance with a practical specific capacity comparable to the theoretical value of 212 mAh g^−1^ [13]. Cathodes based on the polymer **P11** and carbon nanotubes demonstrated a rather high discharge capacity, reaching 165 mAh g^−1^ at the current rate of 0.1C [14]. Sodium cells based on **P12** and **P13** as cathodes provided reversible capacities of 192 and 165 mAh g^−1^, respectively [15]. The covalent organic framework **P14,** used as a cathode in cells with lithium anodes, delivered gravimetric capacities up to ≈100 mAh g^−1^ and good rate capability [16]. The conjugated microporous polymers **P15** and **P17** were utilized as cathode materials in Li cells, providing the discharge capacities of 196.6 and 164.7 mAh g^−1^, respectively, at the C-rate of 0.1C [17]. When cycled in the anode mode in the potential range of 0.01–3.0 V (vs. Li^+^/Li), the polymer **P16** demonstrated an ultrahigh high capacity of 1450 mAh g^−1^, which considerably exceeded the theoretically feasible value [18].

To our knowledge, the highest capacity ever reported for lithium batteries with a stable anthraquinone polymer cathode is 330 mAh g^−1^, which was obtained for poly(dihydroxyanthraquinonyl)sulfide **P4** at a 0.5 C current rate [19]. Its analogue **P3** is one of the most promising cathodes for potassium ion batteries in terms of high reversible capacity (190 mAh g^−1^ at the current density of 20 mA g^−1^) [20].

Here, we report the synthesis of a novel AQ-based carbonyl polymer **PANQ** with a polyaniline core and its detailed characterization as a cathode material for lithium and potassium batteries. The obtained polymer demonstrated record-high discharge capacities, reaching ~360–400 mAh g^−1^ (at 20 mAh g^−1^) in LIBs and ~250 mAh g^−1^ (at 50 mAh g^−1^) in PIBs, in combination with good rate capability and long-term cycling stability. Thus, the introduced polymer **PANQ** can be considered as one of the most promising AQ-based organic electrode materials reported so far for LIBs and PIBs.

The capacities of the most promising organic cathode materials with various redox-active functionalities, such as polyarylamines, nitroxyl radicals, metal–organic frameworks, covalent organic frameworks, etc., have already exceeded 450 mAh g^−1^ in potassium ion batteries [21] and 500 mAh g^−1^ in lithium-based batteries [22]. A comparison of the obtained results with the current state-of-the-art materials demonstrated that **PANQ** was among the best of them in terms of specific capacity. Furthermore, **PANQ** electrodes demonstrated impressive operational stability: 1000 cycles without any notable capacity decay in PIBs.

## 2. Results and Discussion

**Figure 2:**
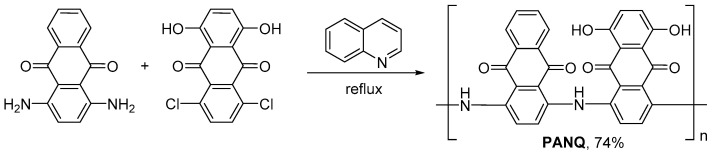
Synthesis of polymer **PANQ**.

Polymer **PANQ** was synthesized using a rather simple approach based on the reflux of a quinoline solution of 1,4-diaminoanthraquinone and 1,4-dichloro-5,8-dihydroxyanthraquinone in an inert argon atmosphere (Figure 2).

The obtained polymer was separated by centrifugation, washed with hydrochloric acid, water, and acetonitrile, and dried in air. **PANQ** was obtained with the yield of 74% as a black powder which was insoluble in common organic solvents and water. However, continuous soaking in the electrolytes led to the partial solubilization of the material (Figure S1). Due to complete insolubility of the material, its characterization was performed using standard solid state techniques, such as elemental analysis, infrared (IR) spectroscopy, solid state magic angle spinning nuclear magnetic resonance (MAS ssNMR) spectroscopy, UV–vis absorption spectroscopy of powders, thermogravimetric analysis (TGA), and scanning electron microscopy (SEM).

**Figure 3:**
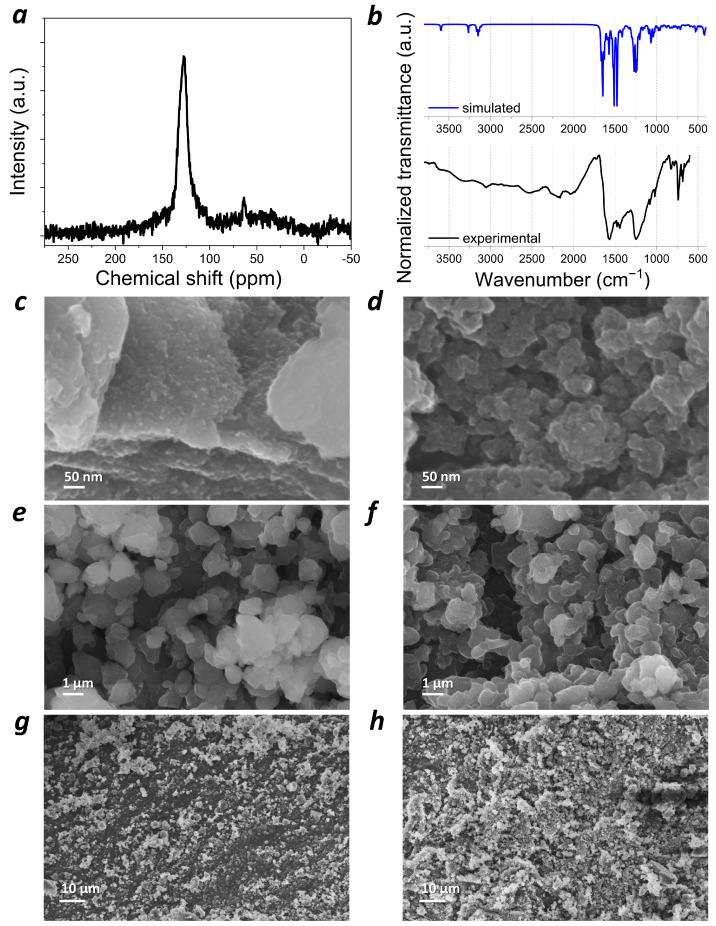
Characterization of the synthesized **PANQ** polymer: (**a**) ^13^C MAS ssNMR spectrum; (**b**) experimental and calculated FTIR spectra; (**c**,**e**,**g**) SEM image of **PANQ** powder before ball milling; (**d**,**f**,**h**) SEM image of **PANQ** powder after ball milling.

The results of C, H, N, O elemental analysis were rather close (C: 69.5%, H: 3.5%, N: 5.0%, O: 17.3%) to the calculated composition of the polymer **PANQ** (C: 70.9%, H: 3.0%, N: 5.9%, O: 20.2%). The ^1^H MAS ssNMR spectrum of **PANQ** (Figure S1a) was not informative since it showed a single broad signal spanning a range of 0–20 ppm. Because the polymerization process did not involve most of the hydrogen atoms in the structures of the starting compounds, the ^1^H MAS ssNMR spectra of the polymer did not differ very much from the spectra of both 1,4-diaminoanthraquinone and 1,4-dichloro-5,8-dihydroxyanthraquinone (Figure S2a). The ^13^C MAS ssNMR spectrum of **PANQ** also revealed a broad signal at 100–160 ppm (Figure 3a). These results are consistent with the chemical structure of the material, which only has aromatic and carbonyl types of carbon atoms. The FTIR spectrum of polymer **PANQ** is rather complex due to the presence of several types of functional groups and different types of bonds in the polymer backbone. It is important to note that the experimental spectrum was in fairly good agreement with the calculated one in terms of both signal positions and intensity (Figure 3b). An analysis of the IR spectra of the precursor compounds and **PANQ** was performed to confirm the molecular structure of the repeating unit. (Figure S3a–c) Both precursors are represented by substituted anthraquinones; so, their spectra are rather similar except for several characteristic bands. For both precursors, groups of signals at 1500–1650 cm^−1^ can be assigned to C=C and C=O stretching, while the signals at 1300–1500 cm^−1^ correspond to benzene ring stretching modes. The low-frequency region features complicated sets of signals and is typically considered as the fingerprint region [23]. However, the IR spectrum of 1,4-diaminoanthraquinone (Figure S3a) had several specific signals, such as 3380 cm^−1^ (corresponding to N-H stretching) and 1403 cm^−1^ (C-N stretching) [23], while the spectrum of the 1,4-dichloro-5,8-dihydroxyanthraquinone (Figure S3b) revealed O-H stretching bands at 3066 cm^−1^, phenolic C-O bending at 1313 cm^−1^ [24], and a strong C-Cl stretching band with the same wavenumber of 772 cm^−1^ as a similar stretching band in 1,4-dichlorobenzene [25]. The IR spectrum of **PANQ** (Figure S3c), as expected, was more complicated than the spectra of the precursors. However, the conversion of all the C-Cl bonds of the precursor compound was confirmed by the absence of a specific stretching band at 772 cm^−1^ in the FTIR spectrum of the polymer. The decrease in the intensity of the N-H stretching band at ~3306 cm^−1^ as compared to that of 1,4-diaminoanthraquinone additionally proves that the polycondensation reaction involves the C-Cl and N-H functionalities of two precursor compounds.

The UV–vis absorption spectrum of the **PANQ** powder was obtained using an integrating sphere. It exhibited wide absorption bands at 400–700 nm, with the tails extending to longer wavelengths, which might point towards the partial doping of this polymer (Figure S4). We also registered the UV–vis absorption spectrum for the lower molecular weight part of **PANQ**, which was soluble in the electrolyte (Figure S4). The **PANQ** polymer demonstrated good thermal stability and did not show any significant thermal decomposition (maintaining ~95% of the original mass) upon heating up to ~350 °C in the nitrogen atmosphere (Figure S5). The DSC results indicated that the decomposition process was endothermic, as expected. The electrical conductivity of **PANQ** was determined as σ = 9∙10^−9^ S/cm, which is close to (6.6∙10^−9^ S/cm, [26]) or lower than (9.5∙10^−8^ S/cm, [27]) the typical conductivity of undoped polyanilines. 

Scanning electron microscopy (SEM) was used to reveal the morphology of the **PANQ** powder. The polymer particles appeared as irregular agglomerates larger than 200 nm in size (Figure 3c), which is why the material was subjected to ball milling in 1,2-dichlorobenzene. This allowed us to significantly decrease the size of the particles down to ~50 nm (Figure 3d).

**Figure 4:**
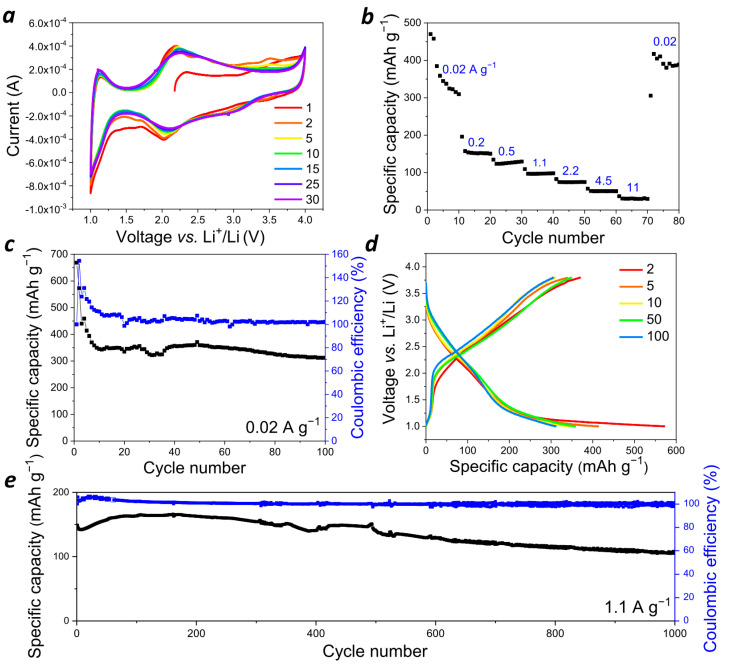
Performance of the Li cells with **PANQ** cathode and 1 M LiTFSI DOL/DME electrolyte**:** (**a**) cyclic voltammogram at 1 mV s^−1^; (**b**) cell rate capability; (**c**) charge–discharge cycling stability at 0.02 A g^−1^ (black line—specific discharge capacity, blue line—Coulombic efficiency); (**d**) charge–discharge curves for selected cycles at 0.02 A g^−1^; (**e**) charge–discharge cycling stability at 1.1 A g^−1^ (black line—specific discharge capacity, blue line—Coulombic efficiency).

To investigate **PANQ** as an electrode material in Li ion batteries, we assembled coin-type cells (CR2032) using lithium as the counter electrode and 1M LiTFSI in the 3:1 (*v*/*v*) mixture of dimethoxyethane (DME) and dioxolane (DOL) as the electrolyte. Li metal has been considered a very promising alternative to the traditional graphite-based anodes due to its ultrahigh capacity (3860 mAh g^−1^) and very low standard negative electrochemical potential (−3.040 V vs. SHE) [28]. The cyclic voltammogram (CV) for the assembled cells registered in the 1.0–4.0 V (vs. Li^+^/Li) potential window. Two broad reduction peaks at 1.8–2.2 V and 3.0–3.3 V and two oxidation peaks at 2.0–2.4 V and 3.4–3.6 V were observed (Figure 4a). Broad peaks in the potential range of 2.0–2.5 V (vs. Li^+^/Li) are typical for polymer materials with redox-active carbonyl groups [16]. The redox transition at higher potentials of 3.4–3.6 V is characteristic of *p*-type polymers such as polyaniline and poly(triarylamines) [29]. To evaluate the contributions of faradaic (diffusion-controlled) and non-faradaic (capacitive) processes, the CV curves were also registered at different scan rates from 1 to 100 mV s^−1^ (Figure S6). Current i (A) depends on the scan rate v (mV s^−1^), as described by the following equation: i = av^b^, where a and b are adjustable coefficients, and the b value accounts for the sum of the faradaic and non-faradaic currents. If only a diffusion-controlled reaction occurs, then the observed current is proportional to the square root of the scan rate, v^1/2^ (Randles–Sevcik equation), while the purely capacitive current linearly depends on the scan rate (b = 1) [30]. The b value calculated for the assembled cells was 0.72, which suggests that both diffusion-controlled and capacitive processes occur in the **PANQ**-based electrodes. 

At the next stage, the charge–discharge behavior was investigated using galvanostatic cycling of the cells in different regimes. The **PANQ**-based electrodes demonstrated the high discharge capacity of ~360–400 mAh g^−1^ at the low current density of 20 mA g^−1^ (Figure 4b–d and Figure S7a). These values are rather close to the theoretical ones calculated for the redox process involving six (342 mAh g^−1^) to eight (456 mAh g^−1^) electrons per one elementary unit of **PANQ**; this means that all of the carbonyl and C-NH-C polyaniline-type groups were involved. With the cycling at low current density, which for organic materials often leads to a fast capacity decay due to the degradation of the active material and/or its dissolution in the electrolyte [31], the **PANQ** cells demonstrated stable operation for least 100 cycles. 

The investigation of the rate capability of the **PANQ** cells (Figure 4b) revealed that, in contrast to the vast majority of inorganic materials, the discharge capacity was reasonable (~80 mAh g^−1^) even at high current densities of up to 2.2 A g^−1^. Continuous cycling at the varied current densities of 0.2, 0.5, 1.1, and 4.5 A g^−1^ also showed rather stable charge–discharge behavior (Figure 4e and Figure S8). For example, the discharge capacity was above 100 mA g^−1^ at 1.1 A g^−1^ for over 1000 charge–discharge cycles, while the coulombic efficiency was close to 100%. Thus, the obtained results clearly show that **PANQ** can be considered a highly promising cathode material for Li ion batteries.

**Figure 5:**
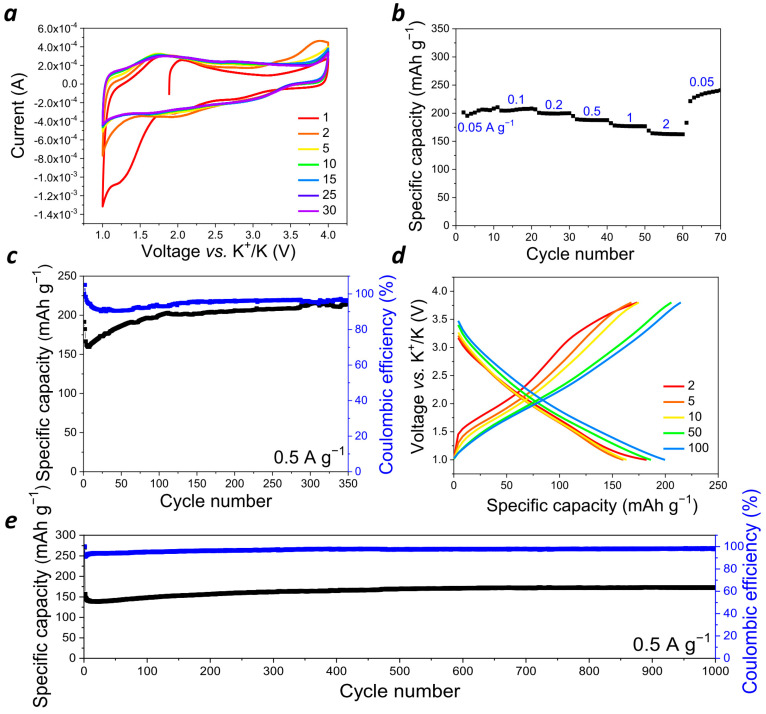
Performance of the cells with potassium anode, **PANQ**-based cathode, and 2.2 M KPF_6_—diglyme electrolyte: (**a**) cyclic voltammogram at 1 mV s^−1^; (**b**) rate capability; (**c**) charge–discharge cycling stability at 0.5 A g^−1^ (black line—specific discharge capacity, blue line—Coulombic efficiency); (**d**) charge–discharge curves for selected cycles at 0.5 A g^−1^; (**e**) charge–discharge cycling stability of the cell with potassium anode, **PANQ**-based cathode, and 1 M KPF_6_—DME electrolyte at 0.5 A g^−1^ (black line—specific discharge capacity, blue line—Coulombic efficiency).

Potassium ion batteries have been proposed as a much cheaper and more available alternative to LIBs since potassium is an abundant element in sea water and the earth’s crust, whereas Li is quite scarce and expensive. Still, conventional inorganic cathode materials for PIBs also suffer from numerous problems, such as low capacity, poor rate capability, and fast capacity decay [32]. However, organic polymers can work as universal host materials to accommodate various metal ions, such as Li^+^, Na^+^, K^+^, and even multivalent ones, such as Zn^2+^, Mg^2+^, and Al^3+^, because of their soft amorphous structure and consequent insensitivity to the radius of inserted metal cations and their very simple redox processes [33]. Therefore, **PANQ** was also explored as a cathode for a potassium battery. Half cells with a metal potassium anode were assembled using 2.2 M KPF_6_ in diglyme (Figure 5a–d and Figure S6b) and 1 M KPF_6_ in DME (Figure 5e and Figure S9). As compared to the cells with the DME-based electrolyte, the batteries with a more concentrated solution of KPF_6_ in diglyme demonstrated slightly better discharge capacities, but their general behavior was quite similar. The cyclic voltammogram (Figure 5a) of the cells with diglyme-based electrolyte in the 1.0–4.0 V (vs. K^+^/K) potential window revealed several broad peaks at 1.5–2.0 and 2.7–3.2 V (vs. K^+^/K). Measuring CV at different scan rates from 1 to 100 mV s^−1^ (Figure S10) delivered the b value of 0.81, meaning that both the diffusion-controlled and the capacitive processes were involved in the cells’ operation mechanism under these conditions.

**Figure 6:**
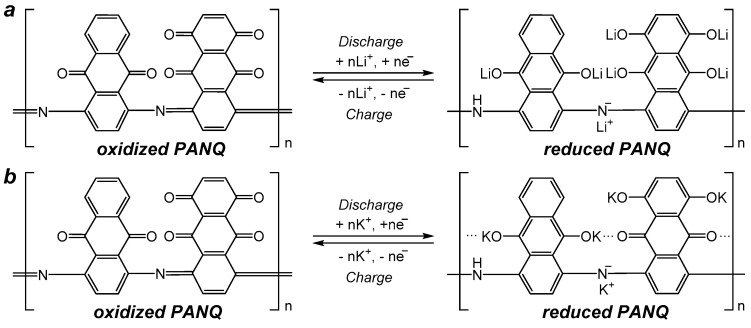
Proposed charge–discharge mechanism for **PANQ** in lithium- (**a**) and potassium-ion batteries (**b**).

Galvanostatic cycling of the cells was performed within a 1.0–3.8 V (vs. K^+^/K) voltage range at different current densities varying from 50 mA g^−1^ to 11 A g^−1^ (Figure 4b–e and Figure S9b,c). It should be noted that the significant increase in the current density (up to 40 times) did not result in the notable drop of the cell capacity (Figure 5b), which demonstrates again the benefits of using organic electrode materials. The cycling at a low current density of 50 mA g^−1^ resulted in discharge capacity values of ~200–250 mAh g^−1^, which means that, on average, four (228 mAh g^−1^) to five (285 mAh g^−1^) potassium ions can be stored per single repeating unit of the polymer. A scheme which presents how up to six potassium ions can be stored in one repeating unit of the polymer is shown in Figure 6. The observed reduction in the capacity of **PANQ** in the potassium cells compared to that of the lithium batteries discussed above is due to the bigger size of the potassium ion, which results in the steric hindrance of the reductive metalation of the neighboring carbonyl groups in this material (Figure 6). 

The potassium cells with **PANQ** electrodes revealed the stable specific discharge capacity of 160 mAh g^−1^ upon cycling at 0.5 mA g^−1^ for over 1000 cycles (Figure 5e) without any decay. The current density of 11 A g^−1^ allowed stable cycling for 3000 cycles (Figure S9c). 

**Figure 7:**
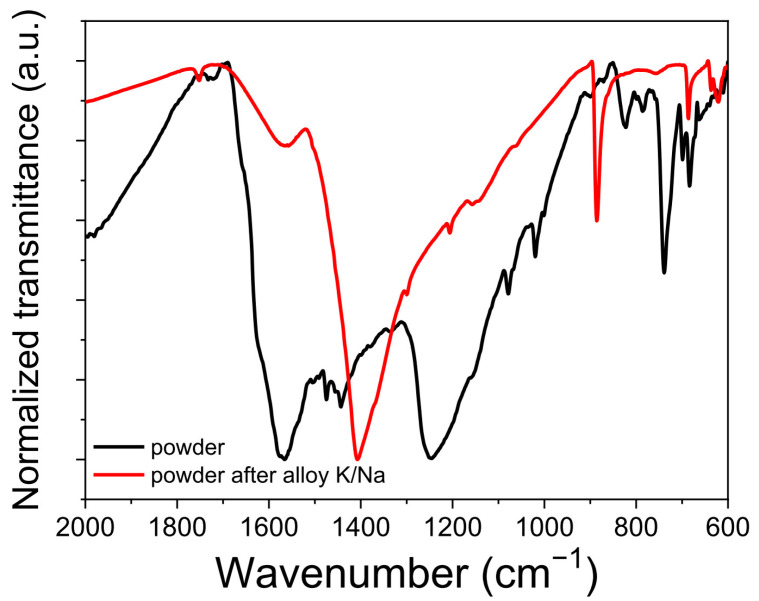
FTIR spectra of **PANQ** before (black) and after (red) metalation with liquid K/Na alloy.

To prove the proposed mechanism of the redox transitions in **PANQ**, we compared the FTIR spectra of pristine **PANQ** and the chemically metalated sample using a liquid K/Na alloy. The changes in the FTIR spectra (Figure 7) confirmed the reduction in carbonyl groups that appeared at ~1600–1700 cm^−1^ in the spectrum of **PANQ** and the emerging of a new –C‒O‒ band at ~1400 cm^−1^ after treatment with the metal alloy, which also concurs with the literature data [34].

**Figure 8:**
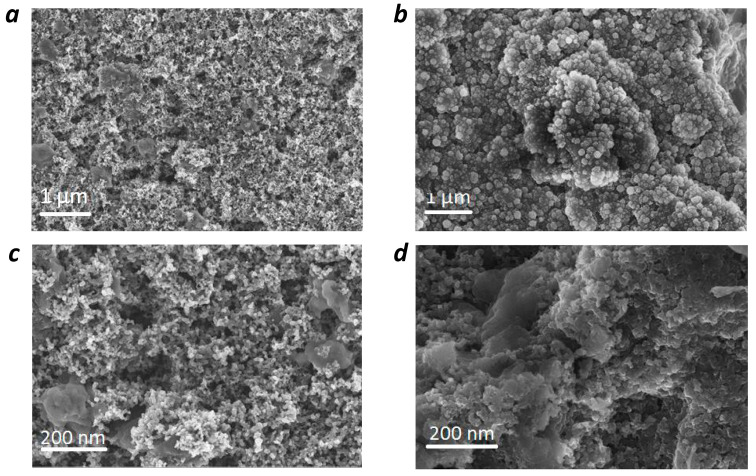
SEM images of the **PANQ** electrode before cycling (**a**,**c**) and after cycling in lithium (**b**) and potassium (**d**) cells.

The cycled cells with both Li and K anodes were opened up and the cathodes were subjected to the post-mortem SEM analysis. Microscopy confirmed the formation of a compact solid electrolyte interface (SEI) layer on the cathode surface in both cases, which could be one of the reasons behind the impressively stable performance of the cells upon long-term cycling (Figure 8). It has been repeatedly reported that conformal SEI formed in situ during cell operation results in improved battery cyclability [35].

**Figure 9:**
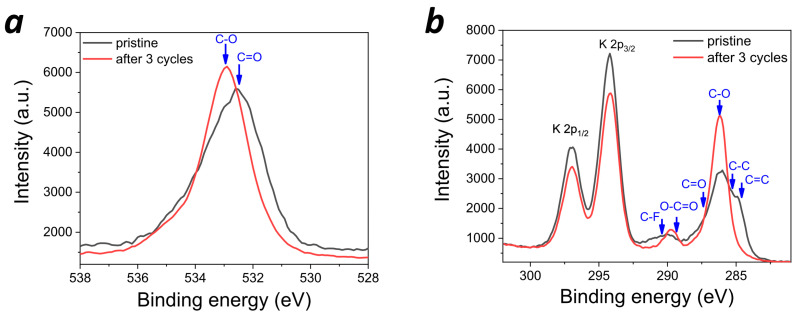
The high-resolution core-level O 1s (**a**) and C 1s/K 2p XPS (**b**) spectra of the **PANQ**-based electrodes in the pristine state and after 3 charge–discharge cycles.

In order to confirm the formation of SEI in potassium batteries, high-resolution core-level XPS spectra were obtained for the electrodes extracted from the freshly prepared batteries and batteries subjected to three charge–discharge cycles (Figure 9). First, the O 1s spectra revealed a high-energy shift, suggesting that the carbonyl groups (C=O) of **PANQ** had disappeared from the surface, while the detected oxygen species were mainly represented by ethers. This is inconsistent with the active material behavior since the cells were disassembled in the fully charged state, which is characterized by the highest C=O group concentration. However, the obtained result could be explained by the electrolyte solvent condensation due to the SEI formation. The changes in the C 1s spectra fully corroborated with this hypothesis. Indeed, the C=C bond signatures of the **PANQ** aromatic core were clearly visible in the C 1s spectrum of the pristine electrode, but then vanished after three charge–discharge cycles. In turn, the C-O ether species dominated on the surface with some minor contributions from either the COO ester or the C-F species at 289.76 eV. The COO groups could have been formed as a result of the deep oxidation of the solvent molecules, while the new C-F signature might have been due to the partial fluorination of the solvent by KPF_6_, which is one of the common processes in SEI formation [36,37]. Thus, the C 1s spectra confirmed that ether solvent condensation products form SEI on the electrode surface. Finally, the comparison of the F 1s spectra (Figure S11) revealed a high-energy shift of the peak corresponding to KPF_6_ due to the change in the surface material composition upon cell cycling (growth of SEI); this was probably also due to the chemical modification of KPF_6_ itself (e.g., replacing some of the P-F bonds with P-O-). Thus, XPS spectroscopy unambiguously confirmed SEI formation on the surface of PANQ cathodes in potassium cells upon cycling. 

To summarize, the anthraquinone-quinizarin copolymer **PANQ** demonstrated high discharge capacities and impressive rate capability and cycling stability in both lithium and potassium batteries. The material presented here represents a highly promising organic cathode that can be easily synthesized from readily available precursors.

## 3. Materials and Methods

### 3.1. Materials

1,4-diaminoanthracene-9,10-dione (Sigma Aldrich, Burlington, MA, USA) and 1,4-dichloro-5,8-dihydroxyanthracene-9,10-dione (TCI, Tokyo, Japan) were used without purification. Quinoline (Acros Organics, Geel, Belgium) was carefully distilled in a vacuum. Anhydrous dimethoxyethane, dioxolane and diglyme, reagent grade N-methylpyrrolidone, 1,2-dichlorobenzene and diethylcarbonate were purchased from Acros Organics (Geel, Belgium).

### 3.2. Synthesis of PANQ

Quinoline (30 mL), 1,4-diaminoanthracene-9,10-dione (0.5 g, 2.1 mmol), and 1,4-dichloro-5,8-dihydroxyanthracene-9,10-dione (0.65 g, 2.1 mmol) were placed in a three-neck round-bottom flask with a magnetic Teflon-coated stirring bar, reflux condenser, stopper, and thermometer. Then, the system was degassed and filled with argon. The reaction mixture was stirred for seven days at 235 °C and then cooled down to room temperature; the precipitate was carefully isolated by centrifugation. The obtained product was washed with quinoline, 5% hydrochloric acid, water, and acetonitrile and dried. The polymer was then purified from low molecular weight fractions using Soxhlet apparatus with diethyl carbonate and dried in a vacuum. Yield: 0.885 g (74%). 

### 3.3. Characterization of PANQ

The Vario Micro cube (Elementar GmbH, Langenselbold, Germany) was applied to perform elemental analysis. A Bruker Avance instrument (400 MHz for ^1^H and 101 MHz for ^13^C) (Bruker, Billerica, MA, USA) using a 3.2 mm MAS probe at room temperature was used to record solid-state NMR spectra. The PerkinElmer Spectrum 100 (ATR) (PerkinElmer, Waltham, MA, USA) was used to register the FTIR spectra. The Simultaneous Thermal Analyzer STA 8000 (Perkin Elmer, Waltham, MA, USA) was used for TGA and DSC analysis (nitrogen atmosphere). The scanning electron microscopy (SEM) images were obtained using a ZEISS LEO Supra25 scanning autoemission electron microscope (Carl Zeiss AG, Oberkochen, Germany). The UV–vis absorption spectrum was registered using Avantes AvaSpec 2048 (Avantes, Apeldoorn, The Netherlands).

### 3.4. Electrical Conductivity of **PANQ**

The electrical conductivity was determined using the Elins P-8 potentiostat (Elins, Chernogolovka, Russia). The current–voltage characteristics (I–V) were recorded at the voltage sweep rates of 10–200 mV/s. Before the measurements, the polymer was pressed into 1 mm pellets, which were placed between stainless steel electrodes. The cell resistance R was calculated according to Ohm’s law: R = U/I, where U is voltage (V), I is current (A), and R is resistance (Ohm). The conductivity was calculated with the formula: σ = d/(R × S), where d is the sample thickness, and S is the electrode surface area (S = 0.2 cm^2^).

### 3.5. Theoretical Calculation of IR Spectrum

The calculations of the IR spectrum of the oligomer with n = 2 were performed using the DFT approach and PRIRODA [38] program package. The molecular structures were optimized using the PBE functional [39] and SBK pseudopotential [40] with the extended basis set C, N, O: [5s, 5p, 2d/3s, 3p, 2d], H: [5s, 1p/3s, 1p] for valence electrons at the Joint Supercomputer Centre of the Russian Academy of Sciences. To broaden the peaks in the IR spectra, the Lorentz form with a fixed linewidth of 20 cm^−1^ was used.

### 3.6. Ball Milling of PANQ

The active material (**PANQ**) was ball-milled using the Fritsch Pulverisette 7 system (Fritsch, Idar-Oberstein, Germany) with zirconia jars and 1 mm zirconia balls. **PANQ** was ball-milled in 1,2-dichlorobenzene for 50 min (10 cycles, 5 min each) at 1000 rpm, and the solvent was then evaporated in a vacuum.

### 3.7. Electrode Preparation

**PANQ** (100 mg) and super C65 carbon (MTI Corporation, Richmond, CA, USA) (80 mg) were thoroughly mixed in a mortar. Then, poly(vinylidenedifluoride) PVDF (Arkema, Colombes, France) (20 mg) was solubilized in 1.5 mL of N-methylpyrrolidone (NMP) and added to the **PANQ**-C65 composite. The resulting dispersion was stirred for 24 h. The obtained slurry was tape-casted on carbon-coated Al foil, dried, and calendered at room temperature. The mass loading of the **PANQ** was in the range of 0.4–0.7 mg cm^−2^. 

### 3.8. Battery Assembly and Characterization

Circular electrodes were cut out (mass loading of the active material of ~0.8–1.4 mg cm^−2^) and CR2032 coin-type cells were assembled in an argon-filled MBraun glove box (MBraun, Garching, Germany). To fabricate lithium coin cells, a lithium disk was used as the counter electrode, and a 1 M LiTFSI solution in a 1:1 (*v*/*v*) mixture of dioxolane and dimethoxyethane was used as the electrolyte (20 µL per cell). A single layer of polypropylene separator (Celgard 2325, 25 µm; Celgard, Concord, NC, USA) was used for the lithium cells.

To fabricate the potassium cells, metallic potassium was pressed onto a stainless steel disc to form the counter electrode, 1 M KPF_6_ in dimethoxyethane or 2.2 M KPF_6_ in diglyme was used as electrolytes (40 µL per cell), and two layers of glass fiber filter (Whatman GF/A Glass microfiber filters, GE Healthcare, Chicago, IL, USA) were used as the separator.

Cyclic voltammograms were recorded with an Elins P40 potentiostat (Elins, Chernogolovka, Russia) at scanning rates of 1–100 mV s^−1^. The galvanostatic measurements were carried out on a Neware BTS3000 station (Neware, Shenzhen, China). The galvanostatic cycling was started from the discharging for all the cells.

### 3.9. *X-ray* Photoelectron Spectroscopy

The cathodes extracted from the potassium cells with 1 M KPF_6_ in dimethoxyethane electrolyte were utilized for the XPS experiments. The XPS spectra were obtained using the PHI XPS 5000 VersaProbe spectrometer (ULVAC-Physical Electronics, Chanhassen, MN, USA) with a spherical quartz monochromator and an energy analyzer working in the range of binding energies (BE) from 0 to 1500 eV. The energy resolution was ΔE ≤ 0.5 eV. The samples were kept in the vacuum chamber for 24 h prior to the experiments and were measured at a pressure below 10^−7^ Pa. All the spectra were calibrated for external reference Au 4f_7/2_ binding energies (84.1 eV).

## 4. Conclusions

We synthesized and characterized a novel redox-active polymer **PANQ** comprising anthraquinone and quinizarin building blocks linked together via a polyaniline-type backbone. When utilized as a cathode in lithium and potassium batteries, the obtained material delivered a high capacity of ~400 and 250 mA h g^−1^, respectively. Furthermore, the **PANQ**-based cells showed impressive rate capability (<20% capacity decrease in potassium cells upon increase in the current density from 0.05 to 2 A g^−1^), which showed the advantages of soft organic materials; these materials are capable of facile metalation and demetallation and can thus enable ultrafast battery operation. In addition, potassium cells using **PANQ** electrodes demonstrated impressive cycling stability: no capacity degradation was observed after 3000 charge–discharge cycles at 11 A g^−1^. Taken altogether, **PANQ** showed the highest performance characteristics among all the known anthraquinone-based redox-active polymers, and therefore, it represents one of the best organic electrode materials for lithium and potassium batteries reported to date. Further rational molecular engineering of quinone-based polymers with a polyaniline conjugated backbone might further enhance the performance of organic LIBs and PIBs.

## Data Availability

The authors confirm that the data supporting the findings of this study are available within the article and its Supplementary Materials.

## Supplementary Materials

The following supporting information can be downloaded at: https://www.mdpi.com/article/10.3390/molecules28145351/s1, Figure S1: **PANQ** in various electrolytes after 4 days, Figure S2: Characterization of **PANQ**: (**a**) ^1^H MAS ssNMR spectra of **PANQ**, 4-diaminoanthracene-9,10-dione (monomer 1) and 1,4-dichloro-5,8-dihydroxyanthracene-9,10-dione (monomer 2); (**b**) ^13^C MAS ssNMR spectra of **PANQ**, 4-diaminoanthracene-9,10-dione (monomer 1) and 1,4-dichloro-5,8-dihydroxyanthracene-9,10-dione (monomer 2), Figure S3: (**a**) FTIR spectrum of 4-diaminoanthracene-9,10-dione (monomer 1); (**b**) FTIR spectrum of 1,4-dichloro-5,8-dihydroxyanthracene-9,10-dione (monomer 2); (**c**) FTIR spectrum of **PANQ**, Figure S4: UV–vis absorption spectrum of **PANQ** powder (red) and soluble fraction of **PANQ** in 2.2 M KPF_6_ in diglyme (black), Figure S5: TGA (black) and DSC (red) curves for **PANQ** powder, Figure S6: (**a**) Cyclic voltammograms at different scan rates for lithium half cells with **PANQ** electrode and 1 M LiTFSI DOL/DME electrolyte; (**b**) the dependence of log(*i*) (current) vs. log(*v*) (scan rate) used for parameter *b* estimation, Figure S7: (**a**) The discharge profile of Li//**PANQ** cell at the first cycle at 0.02 A g^−1^; (**b**) the first discharge profile of K//**PANQ** with cell with 2.2 M KPF_6_—diglyme electrolyte at 0.5 A g^−1^, Figure S8: Charge–discharge cycling of lithium cells with **PANQ** electrode and 1 M LiTFSI DOL/DME electrolyte at different current densities: (**a**) 0.2 A g^−1^; (**b**) 0.5 A g^−1^; (**c**) 4.5 A g^−1^, Figure S9: Performance of the potassium cells with **PANQ** cathode and 1 M KPF_6_ DME electrolyte: (**a**) cyclic voltammogram at 1 mV s^−1^; (**b**) rate capability; (**c**) cycling stability at 11 A g^−1^, Figure S10: (**a**) Cyclic voltammograms at different scan rates for potassium cells with **PANQ** electrode and 1 M KPF_6_ DME electrolyte; (**b**) dependence of log(*i*) (current) versus log(*v*) (scan rate) used for parameter *b* estimation, Figure S11: The high-resolution core-level F 1s XPS spectra of the **PANQ**-based electrodes in the pristine state and after 3 charge–discharge cycles.

## Sample Availability

Samples of the compounds are available from the authors upon request.
