# Supplementary material for: Anthraquinone-Quinizarin Copolymer as a Promising Electrode Material for High-Performance Lithium and Potassium Batteries
Source: Molecules. 2023 Jul 12;28(14):5351. doi: 10.3390/molecules28145351 (PMC10384886; doi:10.3390/molecules28145351)
Supplement: Supplementary file 1 [file molecules-28-05351-s001.zip › molecules-2401770-supplementary.pdf]

Supplementary materials

for the manuscript

Anthraquinone-Quinizarin Copolymer as a Promising Electrode Material for High-performance Lithium- and Potassium Batteries

Elena V. Shchurik [1,2], Olga A. Kraevaya [1], Sergey G. Vasil'ev [1], Ivan S. Zhidkov [3,4], Ernst Z. Kurmaev [3,4], Alexander F. Shestakov [1,5] and Pavel A. Troshin [6,7,1,*]

[1] Federal Research Center for Problems of Chemical Physics and Medicinal Chemistry RAS, 1 Prospekt Akademika Semenova, 142432 Chernogolovka, Russia; okraevaya@inbox.ru (O.A.K.)

[2] Higher Chemical College of RAS, D.I. Mendeleev University of Chemical Technology of Russia, 9 Miusskaya square, 125047 Moscow, Russia

[3] Institute of Physics and Technology, Ural Federal University, Mira 19 str., 620002 Yekaterinburg, Russia; i.s.zhidkov@urfu.ru (I.S.Z.); ernst.kurmaev@gmail.com (E.Z.K.)

[4] M.N. Mikheev Institute of Metal Physics of Ural Branch of Russian Academy of Sciences, S. Kovalevskoi 18 str., 620108 Yekaterinburg, Russia

[5] Faculty of Fundamental Physics & Chemical Engineering, Lomonosov Moscow State University, GSP 1, 1-51 Leninskie Gory, 119991 Moscow, Russia

[6] Zhengzhou Research Institute, Harbin Institute of Technology, Longyuan East 7th 26, Jinshui District, Zhengzhou 450003, China

[7] Harbin Institute of Technology, No.92 West Dazhi Street, Nan Gang District, Harbin 150001, China

* Correspondence: troshin2003@inbox.ru

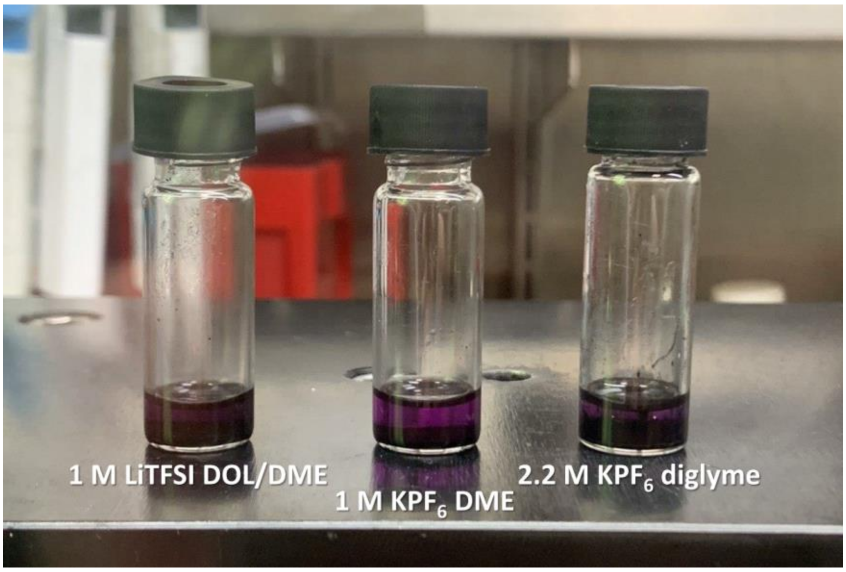

Figure S1. PANQ in various electrolytes after 4 days.

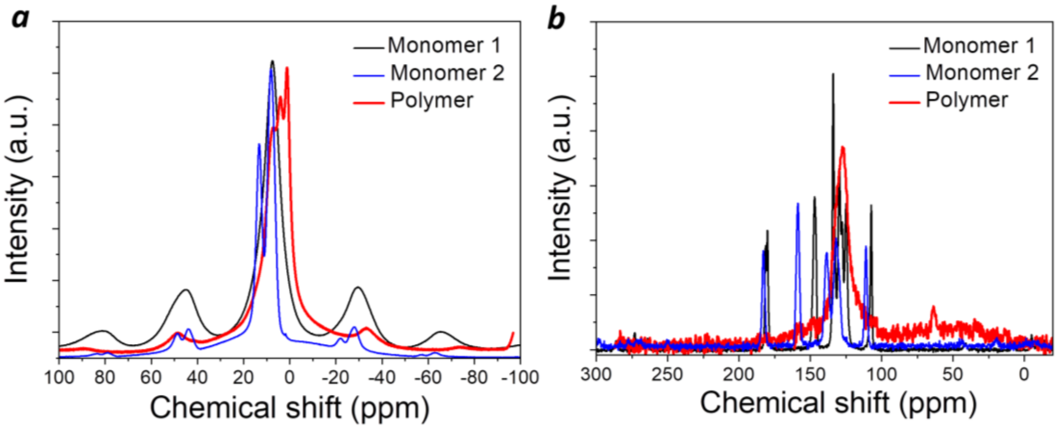

Figure S2. Characterization of **PANQ**: (**a**) ^{1}H MAS ssNMR spectra of **PANQ**, 4-diaminoanthracene-9,10-dione (monomer 1) and 1,4-dichloro-5,8-dihydroxyanthracene-9,10-dione (monomer 2); (**b**) ^{13}C MAS ssNMR spectra of **PANQ**, 4-diaminoanthracene-9,10-dione (monomer 1) and 1,4-dichloro-5,8-dihydroxyanthracene-9,10-dione (monomer 2).

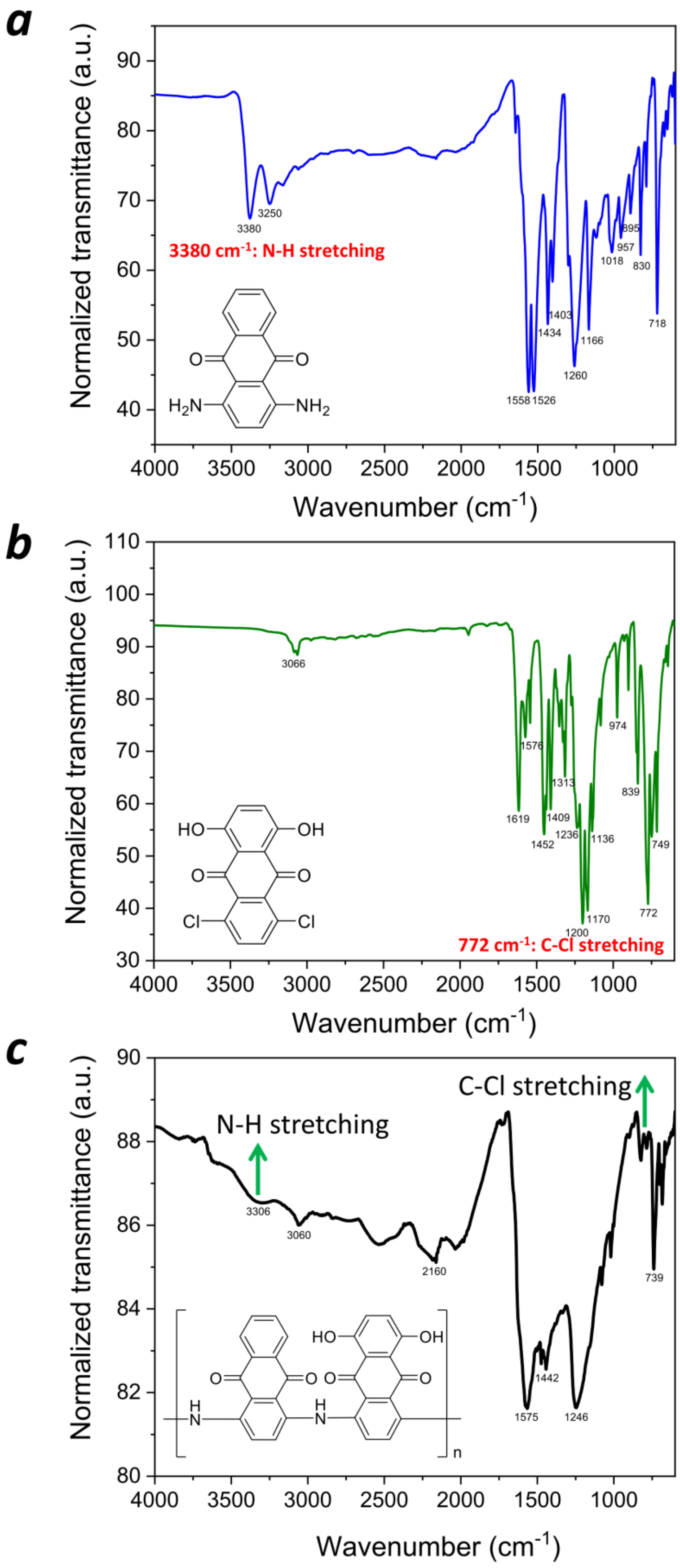

Figure S3. (**a**) FTIR spectrum of 4-diaminoanthracene-9,10-dione (monomer 1); (**b**) FTIR spectrum of 1,4-dichloro-5,8-dihydroxyanthracene-9,10-dione (monomer 2); (**c**) FTIR spectrum of **PANQ**.

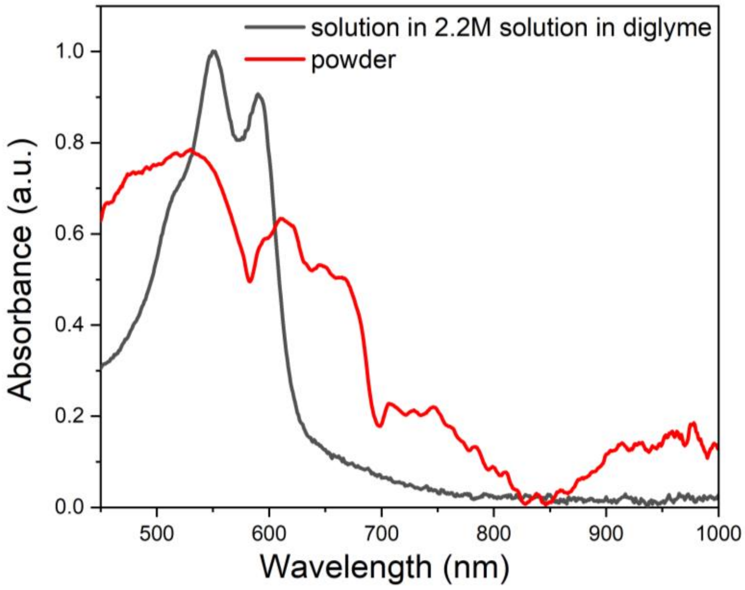

Figure S4. UV–vis absorption spectrum of **PANQ** powder.

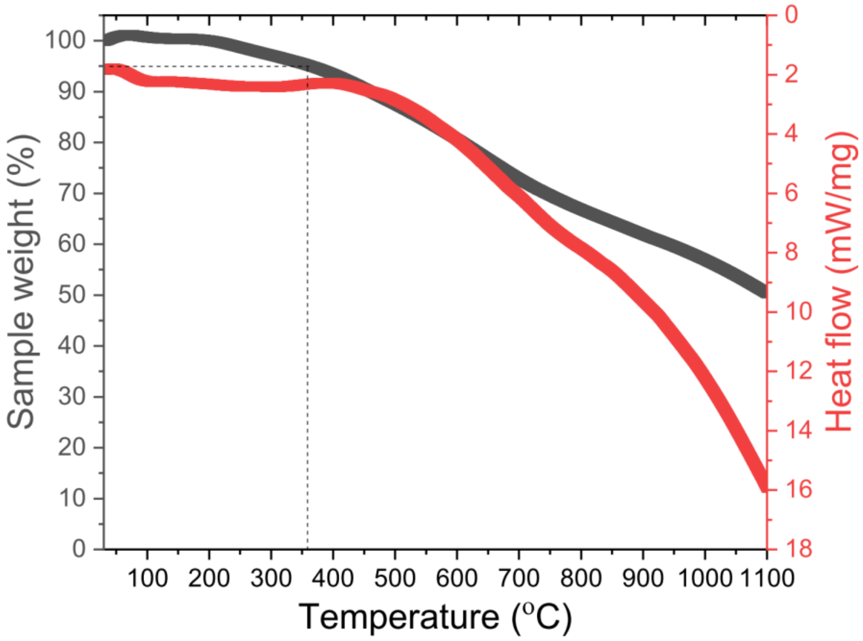

Figure S5. TGA (black) and DSC (red) curves for **PANQ** powder.

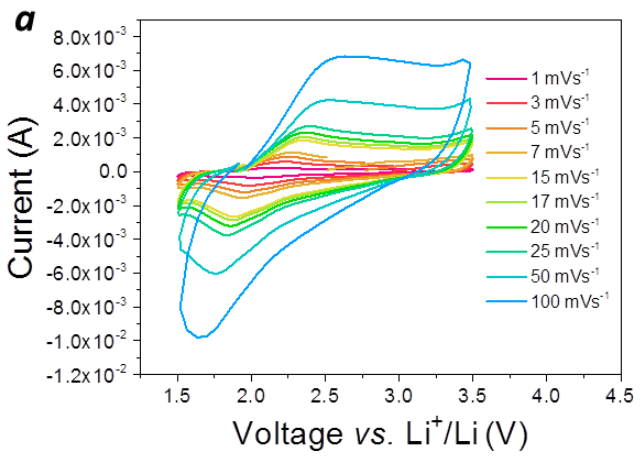

Figure S6. (**a**) Cyclic voltammograms at different scan rates for lithium half-cells with **PANQ** electrode and 1 M LiTFSI DOL/DME electrolyte; (**b**) the dependence of log(*i*) (current) vs. log(*v*) (scan rate) used for parameter *b* estimation.

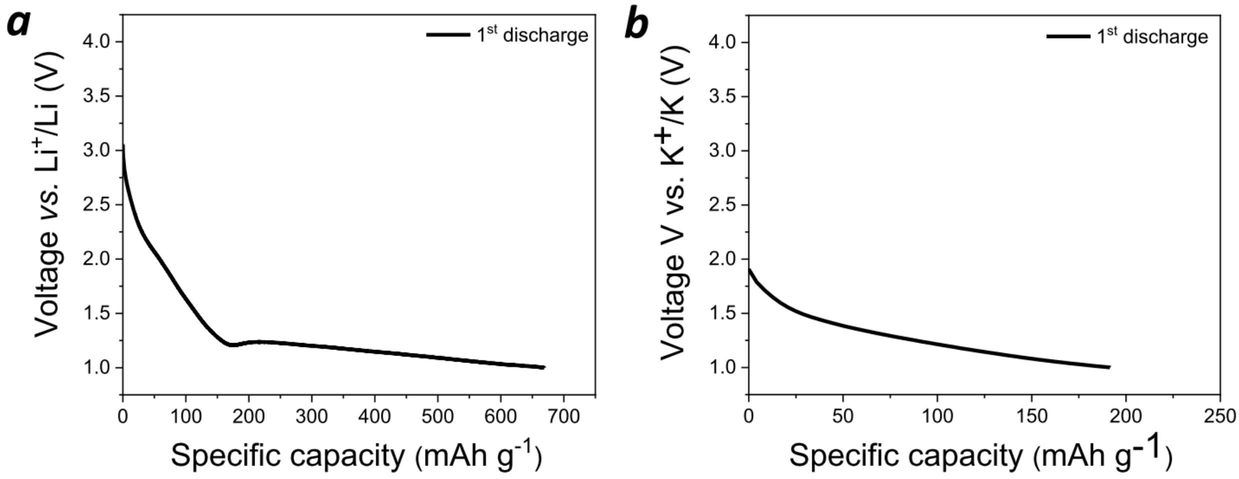

Figure S7. (**a**) The discharge profile of Li//**PANQ** cell at the first cycle at 0.02 A g^{-1}; (**b**) the first discharge profile of K//**PANQ** with cell with 2.2 M KPF_6 – diglyme electrolyte at 0.5 A g^{-1}.

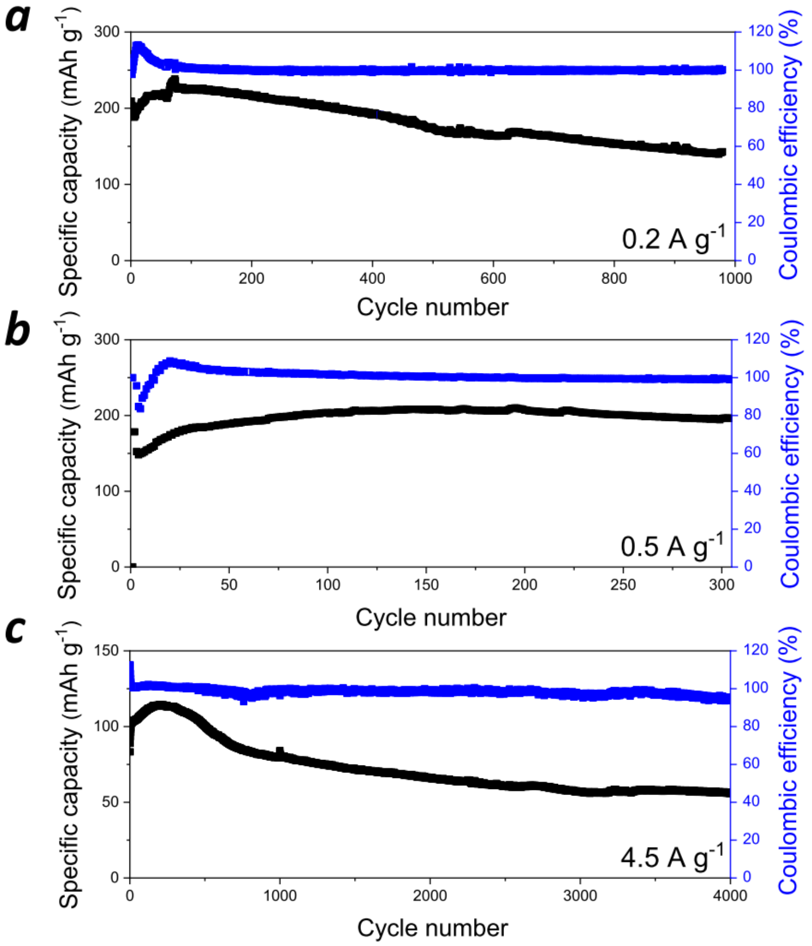

Figure S8. Charge-discharge cycling of lithium cells with **PANQ** electrode and 1 M LiTFSI DOL/DME electrolyte at different current densities: (**a**) 0.2 A g^{-1}; (**b**) 0.5 A g^{-1}; (**c**) 4.5 A g^{-1}.

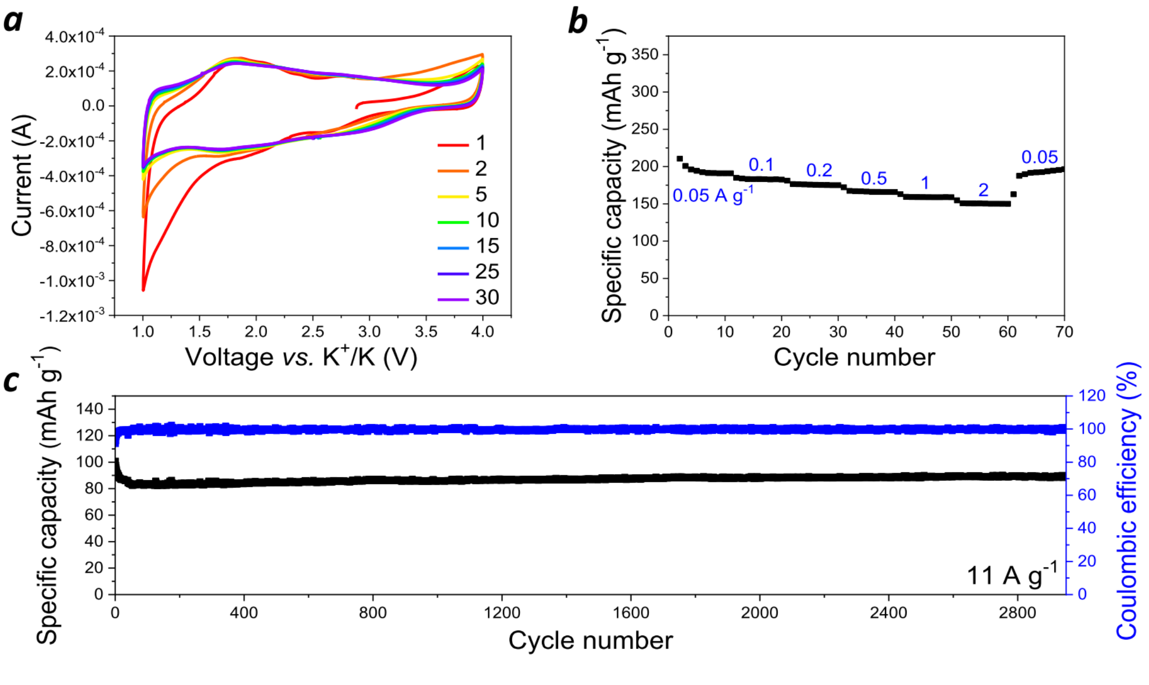

Figure S9. Performance of the potassium cells with **PANQ** cathode and 1 M KPF_6 DME electrolyte: (**a**) cyclic voltammogram at 1 mV s^{-1}; (**b**) rate capability; (**c**) cycling stability at 11 A g^{-1}.

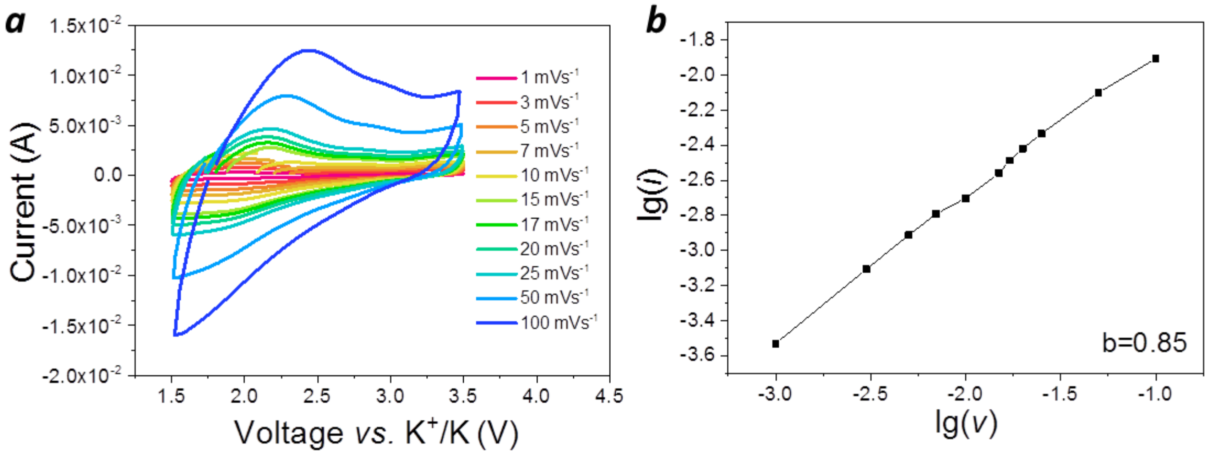

Figure S10. (**a**) Cyclic voltammograms at different scan rates for potassium cells with **PANQ** electrode and 1 M KPF_6 DME electrolyte; (**b**) dependence of log(*i*) (current) versus log(*v*) (scan rate) used for parameter *b* estimation.

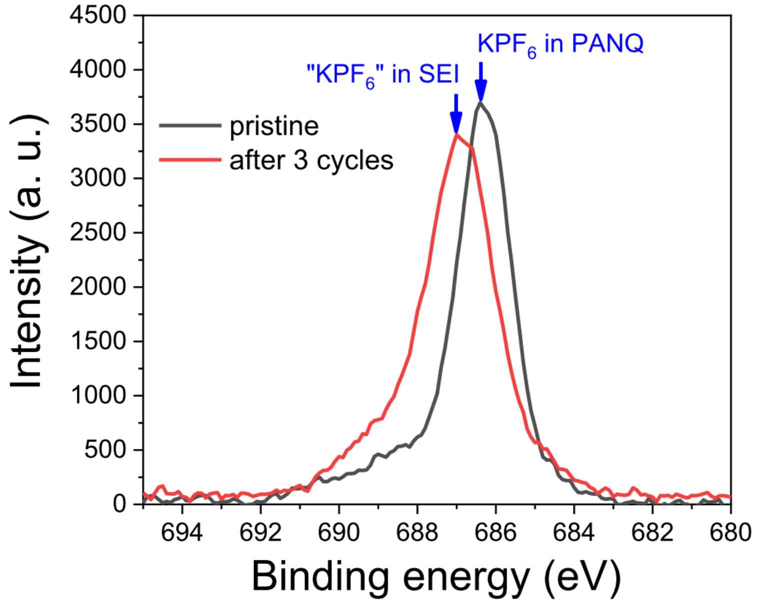

Figure S11. The high-resolution core-level F 1s XPS spectra of the **PANQ**-based electrodes in the pristine state and after 3 charge-discharge cycles.
